# A Novel Quantitative Pain Assessment Instrument That Provides Means of Comparing Patient’s Pain Magnitude With a Measurement of Their Pain Tolerance

**DOI:** 10.14740/jocmr2277e

**Published:** 2015-08-23

**Authors:** Lanny L. Johnson, Andrew Pittsley, Ruth Becker, Allison De Young

**Affiliations:** a 314 East Crystal Downs Drive, Frankfort, MI 49635, USA; b 1430 Cheboygan Ave., Okemos, MI 48864, USA; c 6142 Graedear Trail, East Lansing, MI 48823, USA; d 1784 Alvarado Ave., Walnut Creek, CA 94597, USA

**Keywords:** Pain assessment, Pain, Pain tolerance measurement, Novel quantitative measurement, Patient pain profile

## Abstract

**Background:**

Traditional pain assessment instruments are subjective in nature. They are limited to subjective reporting of the presence and magnitude of pain. There is no means of validating their response or assessing their pain tolerance. The objective of this study was to determine the potential value of a novel addition to the traditional physical examination concerning a patient’s pain and more importantly their pain tolerance.

**Methods:**

Extensive preliminary data were collected on 359 consecutive private practice knee patients referable the subject’s pain, including the magnitude, the most pain ever experienced, and their opinion of personal pain tolerance. The novel evaluation included physical testing of a series of small ball drops through a vertical tube from various fixed levels on the index finger and patella. The patient’s response to this impact testing provided quantitative information, from which a comparison was made to their pain opinion and also to that of other patients with similar demographics.

**Results:**

Nine percent of the patients rated their pain tolerance below the midpoint on the visual analog scale. Seventy-one percent thought they were above the midpoint on the scale in regards to pain tolerance. There were discrepancies in both directions between the subject’s opinion on pain tolerance and their rating of their pain experience to the ball drop testing. Twenty-eight percent of the entire patient group rated themselves above 5 on tolerance, but experienced above the average discomfort compared to other subjects reporting on the finger impact testing.

**Conclusions:**

This report introduces a novel method for collecting data concerning pain that can be subjected to quantification. The database included quantitative measures providing the opportunity to confirm, validate or refute the patient’s assertions concerning pain magnitude and tolerance. This method is best described as a patient pain profile. It has the potential to give both the patient and the physician quantified objective information rendering insight not otherwise available.

## Introduction

Pain is a common presenting symptom and often the chief complaint in almost every clinical practice. It is particularly so with neurological and musculoskeletal problems [1]. The presence of pain and moreover its intensity has an influence on a physician’s clinical judgment, decision making, selection of treatment modalities, potential surgical indications, and the subsequent prognosis. An accurate assessment becomes a major factor affecting decision making in patient management, treatment and outcomes. An understanding of the patient’s perception of their pain intensity and the often overlooked pain tolerance is important in determining a rehabilitation schedule and the return to work or activities.

However, existing clinical pain assessment instruments are restricted to the patient’s subjective perception of intensity. The numerical rating scale, verbal descriptor scale, visual analogue scale, and other methods are solely dependent upon the patient’s opinion [2-8]. These assessments are subjective by nature. There is no assessment of pain tolerance in the traditional methods. These pain clinical evaluations are lacking the quantitative validation and confirmation found in other aspects of clinical practice.

By way of contrast in cardiology, the complaint of an irregular heart beat can be assessed with a stress EKG. In neurology, a numb extremity may be evaluated by topical two point discrimination testing. In orthopedics or rehabilitation medicine, angulation deformity and range of motion are measured in degrees. Limb lengths, joint circumferences and ligamentous instabilities are recorded in millimeters. These quantitative clinical measurements provide an objective means of confirming, validating or refuting the patient’s specific symptoms. Such objective findings are considered important in individual patient care and are used not only in making clinical judgments, but to create surgical indications, treatment algorithms, establish practice standards, clinical trials and outcome measurements [9]. However, the traditional visual pain assessment is lacking any opportunity for confirmation or validation. The physician is restricted to accepting the patient’s subjective opinion of this single factor, pain intensity.

Pain assessment and management is recognized with increasing importance in patient management. In January 2001, the Joint Commission on Accreditation of Healthcare Organizations mandated pain assessment for its member institutions [10]. This document included the statement, “Patients have a right to appropriate assessment and management of pain”. Subsequently, pain assessment was considered important enough to be routinely documented as the fifth vital sign. About that time and since there have been a number of publications concerning pain, but few in the orthopedic literature concerning assessment measurement [11-22]. Existing pain assessment tools are typically a combination of pain specific questionnaires providing for self-reporting limited to the present intensity of pain recorded on a horizontal analog scale or similar method [2-9]. The use of word “tools” in this context should not be mistaken for the customary use in medicine where tools are reserved for some type of physical instrument or machine to perform a task.

Various attempts have been made to quantify pain with use of instruments for touching, pinching, cutting, and application of heat and cold [23-27]. Tests that included burning or lacerations do not seem suitable for a clinical practice. When a cut, cold or burn is used, the method has potential problems with standardization and/or calibration of the instrumentation. Other assessments have included the natural pain of child birth as a control [28, 29]. In 2007, the NIH funded the development of a new pain assessment instrument [30]. This “instrument” is psychologically based, subjective in nature and primarily intended for research purposes. There remains difficulty in assessing a patient’s pain because of the subjective nature and the limited related information. The existing pain assessment tools provide no objective measure or practical clinical means for a benchmark comparison of the subject’s opinion of their pain, their pain tolerance or a comparison of their pain experience to anything or anyone else [2-7, 9, 16, 23-29, 31, 32].

Traditional pain assessments instruments document only the patient’s opinion of the magnitude of their present pain [2-8]. The patient’s opinion is typically accepted without verification and there is no component to confirm or refute the assertion of pain magnitude. The common use of the word assessment in reference to pain instruments is contrary to the accepted definition. The free medical dictionary states: “patient’s subjective report of the symptoms and course of the illness or condition and the examiner’s objective findings, including data obtained through laboratory tests, physical examination, medical history, and information reported by family members and other health care team members.” [30] (http://medical-dictionary.thefreedictionary.com/assessment).

Notice the wording “the examiner’s objective findings” is absent in the typical pain assessment [2-8]. Modern day accepted clinical practice assessments subject the patient’s subjective symptoms to verification. The standard clinical evaluation report includes a statement that the patient’s symptoms were verified or they were not confirmed by additional related testing. However, when it comes to pain assessment, there is no further testing to confirm or refute the presenting clinical subjective complaint.

With present day economic mandates for limited hospitalization and outpatient surgery, pain management has become an important issue [33, 34]. It is an important issue in palliative care [35]. The presence and magnitude of pain is important, but even more important in patient management is the consideration of the individual patient’s pain tolerance.

**Figure 1:**
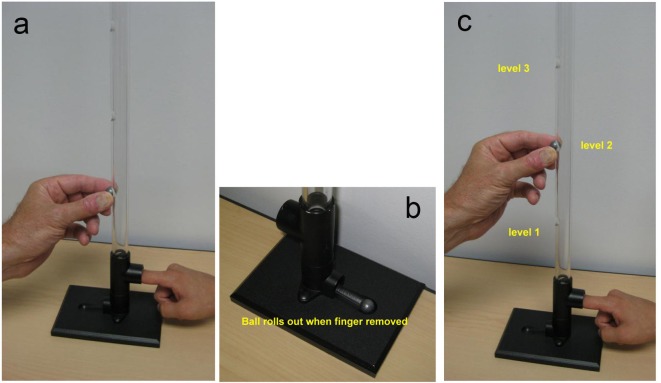
Photographs of the instrument used for finger impact testing. Notice patient’s index finger in the tube prepared to verbally respond to the experience of the impact’s intensity; 0 to 10 in magnitude. The subject’s eyes are closed so as to isolate the experience to the physical impact of the ball drop. After the ball drop the person removes their finger and ball drops out. Person’s finger is replaced in the tube for the next test. (a) Ball drop from the lowest portal to initiate the testing. (b) Close up of the base with portal for the subject and ball retrieval. (c) Examiner releasing ball drop on subjects finger nail in the instrument.

**Figure 2:**
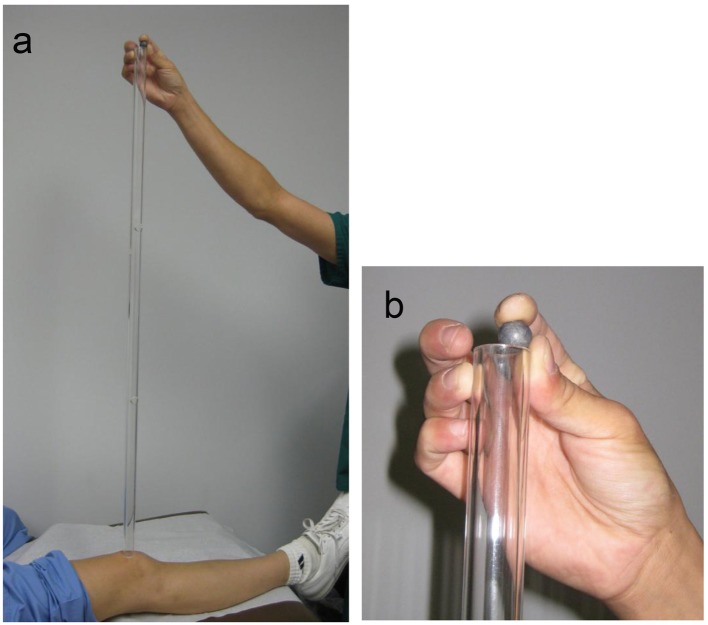
Photograph of the 3-feet long tube used for knee patellar impact testing. (a) Examiner is placing the ball at the highest of the three 1-foot openings in the 3-feet tube. The subject’s eyes are closed so as to isolate the experience to the physical impact of the ball drop. All subjects and patients permitted the ball drop at this highest level. There are openings at 1 and 2 feet and the top. (b) Close up of examiner placing ball in top portal.

This report introduces a novel, simple, practical, inexpensive, efficient, validated clinical method of expanding the concept of pain assessment, so that it joins the list of other quantifying clinical measures. The method is initiated by recording three subjective factors. There is the patient’s perception of their present pain intensity. The second factor is unique: the recording of the patient’s opinion of their personal pain tolerance. The third factor is the patient’s response to a validating physical impact test. This test is performed with an instrument consisting of a tube into which a small lead ball is dropped from measured distances upon the affected body part (Fig. 1, Fig. 2). The physical impact testing provides an opportunity for validating what the patient perceives about their pain intensity and tolerance. The responses to these three factors plus the medical history and clinical diagnosis are entered into a computerized database for subsequent quantitative analysis.

The database includes patients of similar demographics and diagnosis. Mining of the database provides validation of the patient’s opinion of their pain intensity and pain tolerance with the impact testing results. In addition, there is the opportunity to compare the patient’s results to similar cohorts by demographics and clinical impression.

This method seemed best characterized by the term patient pain profile considering the quantitative nature of the abundant data collected, collated, and compared. The use of the word profile was defined medically as “a summary representing quantitatively a set of characteristics determined by tests” [30].

The purpose of this report is to introduce an expanded pain assessment instrument that fulfills the accepted definition and includes testing to verify the patient’s pain magnitude as well as their pain tolerance. The expectation was that use of this patient pain profile in this proof of principle pilot study would demonstrate its potential value in patient management and care.

## Material and Methods

In 1990, this novel addition was made to the routine physical examination in the private practice of the senior author. A series of minimal impacts were made on the patient’s index finger and patella. The magnitude of the impact was similar to that created during testing the patellar tendon for neuromuscular reflex with a rubber mallet percussion hammer. The data were collected on 1,000 consecutive patients with various presenting orthopedic complaints between November 5, 1993 and February 28, 1995. In addition, the physical examination test was performed on two study groups: one was a study group with historically asymptomatic knee and the second was patients presenting with knee problems [36]. Subsequently, this existing de-identified patient database was mined to assess any potential clinical value to the novel method of impact testing. The result is the subject of this report, the patient pain profile.

This after the fact study was performed under the exemptions listed at §46.101 a (4) in the Code of Federal Regulations; Title 45, Public Welfare, Department of Health and Human Services, part 46, Protection of Human Subjects [PDF 215 kb]; Revised January 15, 2009, Effective July 14, 2009. It states “While IRBs can be more inclusive or restrictive, under the statute, exemptions to IRB approval include research activities in which the only involvement of human subjects will be in one or more of the following categories: Research involving the collection or study of existing data, documents, records, pathological specimens, or diagnostic specimens, if these sources are publicly available or if the information is recorded by the investigator in such a manner that subjects cannot be identified, directly or through identifiers linked to the subjects.” [37] (http://www.hhs.gov/ohrp/humansubjects/guidance/45cfr46.html#46.101).

A proprietary office-based electronic medical record facilitated the study (Benevolent Dictator^®^ Information Health Network, Okemos, MI, USA).

### Group 1

This group consisted of asymptomatic subjects who were from a previous reported study of assessment of people who never had a knee problem [36]. There were men (n = 129) and women (n = 64) in this group. The average age of the men was 48.4 years with range of 21 - 85 years. The average age of the women was 51.2 years with range of 25 - 84 years.

### Group 2

This group consisted of symptomatic patients presenting with knee problems examined in the senior author’s private practice. There were men (n = 204) and women (n = 155) in this group. The average age of the men was 42.3 years with range of 10 - 89 years. The average age of the women was 42.4 years with range of 9 - 84 years.

The women subjects in group 1 were seen several months prior to this study and were requested to return for the impact physical exam test [36]. Sixty-four of the 100 women returned. The novel physical exam finger and knee impact testing on the male gender group was preformed at the time of the initial evaluation and examination [36].

After patients filled out the standard medical history forms, they were given an explanation and demonstration of the novel finger and knee impact physical examination. They were given the option to accept or decline. If they agreed, they were given the pain-specific questionnaire developed for this study (Supplementary 1, http://www.jocmr.org). The separate cohort of subjects who never had knee symptoms had a similar introduction. Upon the subjects acceptance, the additional questionnaire was presented that had expanded pain specific questions. Both groups were to write down whether they had pain of any type or place at the present time. They were to indicate the most pain they ever experienced and if they were presently taking pain medication. They were then to rate their present pain on a 0 to 10 horizontal pain scale, with zero being no pain at all, and 10 being the most pain that they could imagine. Unique to this pain assessment method was the request for the patient or subject to rate their pain tolerance on a visual horizontal numerical analog scale of 0 to 10, with zero being no pain tolerance at all, and 10 being the most tolerance that they could image. The subject’s medical record, demographics, and the responses to the impact testing were entered into a cumulative database of the electronic medical record in an anonymous manner to protect the subject’s privacy.

The impact testing was performed with a physical instrument in the traditional sense (Fig. 1, Fig. 2). It was devised by the senior author and provided a method to measure the person’s response to a series of physical impacts of various magnitudes. The physical instrument consisted of two devices: one for testing the finger nail and the other for the knee (Fig. 1, Fig. 2). The instrument used a small lead ball drop to create the physical impact. The lead ball is sold in most gun shops as a 0.54 caliber rifle round ball. It is 0.53 inch in diameter and weighs 14 g. The device for the finger nail testing consisted of a platform with a 2-feet high transparent plastic tube assembled on top of the platform (Fig. 1). This tube was clear plastic with outside diameter of 7/8 inch and inside diameter of 9/16 inch. The tube has perforations at 4-inch intervals for placement of the lead ball. The platform has a place for the patient to place their finger. The device for testing the knee was a plastic tube of same diameter, 3-feet high with perforations at 1-foot intervals (Fig. 2). Gravity provided the standard force from the various uniform heights.

To allay any apprehension, the patients were shown the instrument and how the test would be performed. They were encouraged to handle the lead weight and perform a free drop on their own fingernail from various short distances. They were advised that they could refuse the test or conclude the test at any time for any reason, especially if it was too painful. During subsequent formal testing, the patient was asked to have their eyes closed so as not to see the level from which the ball was dropped. The patient was instructed to give a response to each impact of 0 to 10 with zero being no pain or discomfort and 10 being the most pain they could imagine. This was the same spectrum of responses used for their tolerance reporting.

The patients were subjected to a series of small lead ball drops on the nail of the index finger of each hand from ever increasing higher standardized distance starting at the lowest level and advancing upward (Fig. 1). The extent of the upward progression was to be limited by the patient’s tolerance or their request to stop for any reason. The lead ball drop test was then repeated upon each patella through the 3-feet tube at a distance of 1, 2 and 3 feet (Fig. 2). The tests were repeated three times at each level to assure consistency and reliability. One person, a licensed practical nurse (LPN) performed all the tests in this study and recorded the data. At the conclusion, the patients were asked their opinion of the test. Their responses were recorded on the form and placed into the computerized database (Supplementary 1, http://www.jocmr.org).

In clinical practice, upon completion of the patient’s testing, the results were entered in a database and displayed on a computer screen for review simultaneously by the patient and physician. The computer display provided a format for discussion on findings of consistency and/or inconsistencies between the patient’s opinion of pain tolerance and their rating of the pain experienced by the impact test. There was also the opportunity to discuss their personal results compared to others in the database of similar demographics and diagnosis. The physician offered no opinion or advice until after the patient had independently processed the information in regards to their possible treatment choices.

The midpoint on the numerical visual analog scale of 5 was arbitrarily determined to be the middle value (not the actual mean or median) for the purposes of analyzing the data in this study. It was presumed the patient would interpretate the midpoint was perhaps the mean or median for reporting their responses. It was logistically the midpoint on the scale so the subject would probably assume this to be the mid value (Supplementary 1, http://www.jocmr.org). Therefore this arbitrarily determined midpoint of 5 was used to assign those above and below this position on the scale for rating of pain and pain tolerance (Supplementary 1, http://www.jocmr.org). Actual calculations of mean and median were also made from the data.

In the clinical groups, a comparison was made on the experience of the patellar impact test between the patient’s symptomatic and the asymptomatic knee. There were 287 patients who had this clinical situation, meaning the other knee was asymptomatic. Bilateral cases of necessity were excluded. Since all patients allowed patellar impact testing to progress to the top level of 3 feet, only the top value was used in the calculations.

Validation of this pain assessment instrument was performed on a subset of the subjects, the asymptomatic men group. This was intended to test the consistency of initial responses to those of a subsequent time. The subjects were not advised of the probability of the request for the second test. The 113 men with asymptomatic knees were requested by mail to return for the second pain assessment test. Thirty-one of the 113 returned at an interval of 1 month for the second test.

Data were analyzed using SPSS version 13.0 (Chicago, IL). After checking for normal distribution of the data, an independent *t*-test was used to analyze the difference in self-reported pain tolerance between groups. The Mann-Whitney U test was used to analyze the difference in experienced pain during the test between groups. To establish a group’s mean finger pain experienced during the test, the mean pain experienced in the right and left fingers at the top level was calculated. Nonparametric Wilcoxon signed ranks test was used to analyze the difference in self-reported pain tolerance and pain experienced within each group. All statistical tests were two-tailed and the alpha level was set at 5%.

## Results

The knee patient’s self-reporting of their present pain intensity showed a spectrum of responses from 1 to 10. The mean was 6.6 and the median was 7. As expected the general present pain intensity ratings were lower within the asymptomatic knee study groups.

### Rating personal pain tolerance

Using the arbitrarily determined midpoint of 5 as the bench mark on the numerical visual analog scale, 9% of the entire cohort rated their pain tolerance below 5, presumably below average rating. Twenty percent selected their tolerance rating at 5. Seventy-one percent thought they were above the midpoint on the scale in regards to pain tolerance.

The median rating of personal pain tolerance on the horizontal visual analog scale was the slightly different for subjects in the study groups and the clinical patients. The mean pain tolerance for the asymptomatic study groups was 6.8 (standard deviations of 1.5). Asymptomatic men self-reported their pain tolerance at a mean of 6.6 (range, 2 - 10) and asymptomatic women 7.2 (range, 2 - 10).

The mean pain tolerance rating recorded by the symptomatic patients was 6.5 (standard deviation of 1.7): symptomatic men 6.6 (range, 1 - 10) and symptomatic women 6.3 (range, 1 - 10).

The asymptomatic study group reported higher pain tolerance than the patients. There was a significant difference in the self-reported pain tolerance between groups (P = 0.04). However, there was no significant difference in the pain experienced during the finger impact test between groups (P = 0.97) (Table 1).

**Table 1:** Comparison of Pain Tolerance and Pain Experience Between Groups on Finger Testing

Variable	Asymptomatic group mean (SD)	Symptomatic group mean (SD)	P-value
Self-reported pain tolerance	6.7 (1.5)	6.5 (1.7)	0.04
Pain experienced during test	3.2 (2.2)	3.4 (2.6)	0.97

3 = height of 12 inches. The asymptomatic study group reported a significant higher pain tolerance than the patients. However, there was no significant difference in the pain experienced during the finger test between groups (P = 0.97).

### Discrepancies in opinion on pain tolerance versus the testing experience

There were some discrepancies between the subject’s opinion on pain tolerance and their rating of their pain experience to the ball drop testing. The discrepancies were in both directions, higher and lower. Twenty-eight percent of the entire patient group rated themselves above 5 on tolerance, but experienced above the average pain or discomfort compared to other subjects reporting on the impact test. They experience pain or discomfort at a lower height of ball drop than other subjects who took the test. Those patients in this “sensitive” group had the following diagnoses: degenerative joint disease (DJD) 38; torn medial meniscus 27; torn anterior cruciate ligament (ACL) 25; torn lateral meniscus 12; patellar dislocation 5; patellar degenerative joint disease 5; osteochondritis dessicans 5; torn tibial collateral ligament (TCL) 3; anterior knee pain 3; pseudogout 3; contusion 3; chondromalacia patella 3; post-operative anterior cruciate ligament 2; Baker’s cyst 2; patellar subluxation 2; loose bodies 2; torn PCL 1; patellar fracture 1; normal 1; Osgood-Schlater’s 1; tendonitis 1; psoriatic arthritis 1; gout 1; loose tibial total knee component 1.

In the other direction of inconsistency, 3% said they had pain opinion tolerance less than 5 and experienced less than the average pain or discomfort of other subjects to the impact test. Those patients in the clinical group that estimated their pain tolerance below 5 coupled with below the average pain experience to the finger impact test had the following diagnoses: DJD 5; ACL 4; torn medial meniscus 3 and one each of torn TCL, osteonecrosis, patella baja, subluxation patella, dislocation patella, and contusion.

### Knee impact test

Since all subjects tolerated the test to the top 3-feet level, this data point was used for the comparison. The symptomatic patients experience slightly more pain than the asymptomatic subjects. Patients with one symptomatic knee were assessed comparing the pain experience on the symptomatic knee and the asymptomatic kneecap with the impact test. The mean pain experience to the ball drop test was at reported at 2.9 feet for the symptomatic knee and 2.5 feet for the opposite asymptomatic knee.

There were 541 in all (symptomatic clinical patients and asymptomatic study groups) with valid results for comparison of their opinion of pain tolerance to their pain experience with the knee ball drop test. Seventy-four percent (n = 402) held the opinion that their pain tolerance was above 5 on a scale of 0 to 10. However upon knee experience testing, 59.4% (n = 239) of the high tolerance opinion group recorded below average pain experience on ball drop test. In other words, most (74%) of all those undergoing the knee test stated they had above normal pain tolerance, but only 43.5% (n = 235) of them confirmed it by the experience. Sixty-nine percent of men had rated themselves as higher than the midpoint of 5 (6 - 10) on pain tolerance. However this high opinion tolerance group (78%) of men experienced greater pain than the average. Sixty-nine percent of the women rated themselves on pain tolerance higher than the midpoint of 5 (6 - 10), but 77% of this group state they experienced more than the average amount of pain on the knee impact test. Like the composite data, all subgroups showed similar discrepancies of over evaluating their pain tolerance compared to their pain experience with the impact knee test.

### Test method validation

Thirty-one men from the asymptomatic knee study returned for validation of their responses. They initially self-reported the estimation of pain tolerance at 6.6 average. Upon return, the average score was 6.8 on the unanticipated second test. They were consistent on the pain experience testing at both time intervals. They showed a 3.1 average pain experience at the highest level of ball drop on the finger on both occasions.

This expanded assessment testing required an average 5 min of the examiners time. The impact of the ball drop was tolerated well by patients on both index finger nails and the knees and without injury. Most subjects allowed testing to the top height on both finger and knees. At the top level, the pain experience on the finger nail impact was rated at an average of less than 4 on a scale of 10. The knee impact test experience average was less than 3 of 10 at the top level. The testing did not approach the level of pain intolerance for any subject, but still provided enough data points to evaluate the spread of responses.

The participant’s responses at the conclusion of the test were favorable. Ninety-eight percent said they liked it, 2% disliked it and no one filled in the blank area inviting suggestions (Supplementary 1, http://www.jocmr.org).

## Discussion

The potential of this patient pain profile as a valuable clinical assessment method was demonstrated in this proof of principle pilot study. This method expands the conventional physical examination to include a pain assessment beyond subjective perception to objective physical examination quantitative measurements. The results and analysis provided clinical insights not otherwise disclosed. The instrumentation is inexpensive and may be personally constructed. The method is not time consuming. It was clinical practice friendly. The patient acceptance was high.

This patient pain profile overcomes the limitations of existing clinical pain assessment “instruments” that remain subjective in nature and are predominantly psychological in design. Existing pain assessments are limited to accepting the patient’s subjective perception of their pain intensity. The patient’s opinion is the sole factor contributing to the assessment [2-9]. There is no quantitative analysis or meaningful comparisons to a benchmark cohort [2-9]. The result does not increase the patient awareness or produce any clinical insight [2-9].

Although this study cohort consisted only of knee patients from a musculoskeletal clinical practice, this patient pain profile has broad clinical application. Patient perception of pain intensity and pain tolerance are not necessarily condition specific. The use of the finger nail for impact testing is suitable as a universal standard independent of the medical condition or diagnosis.

The expanded pain clinical evaluation introduced herein is multifaceted and beyond the limited information used in most pain assessment instruments. It records the patient’s present pain intensity, their opinion of personal pain tolerance, and the results from the measured physical impact experience. The information is entered into a computerized data base. The patient learns if there were any inconsistencies between his/her opinion of pain tolerance to the impact experience. Their results are available for comparison to others of similar demographics and diagnosis. The method provides individualized personal insights not otherwise available concerning the patient’s pain. This method produces a patient specific pain profile. This method qualifies as a medical profile which is defined as a summary representing quantitatively a set of characteristics determined by tests [30].

The strength of this study was the introduction of a physical testing instrument into the traditional physical examination that provides a quantitative measurement. This is contrasted with pain assessment “tools” that are limited to verbal or written responses concerning present pain [2-7] (Fig. 1, Fig. 2). The impact method for pain experience testing closely represents mechanisms of common physical origin: injury, fall, hit, force, push, twist, jumping, walking, and running. The testing method was always below the threshold of producing an experience of objectionable pain, yet provided a spectrum of data points for the various comparisons. Importantly the expanded nature of the method provided opportunity to compare the patient’s self reported pain tolerance with their quantitative test pain experience. Additional insight was possible with comparisons of the subject’s responses to others of similar gender, demographics and diagnosis. Importantly, this expanded pain assessment instrument allows the patient to be in control of reporting all the data. The health care provider is only recording the patient’s experience, collating and sharing the information. The sensitive nature of the patient’s compliant of pain is discussed solely on the basis of the patient’s input, absent any examiner or physician bias or prejudice. Perhaps this non-threatening aspect of the pain evaluation contributed to the 98% favorable response to the method.

There are several weaknesses in this report. Although the existing clinical practice database includes over 1,000 orthopedic patients, the future use of this pain assessment instrument should include more patients from a broader demographic group: age, genetic heritage, geographic location, worker’s compensation, third party liability. This report focused only on the novel nature of the pain assessment method comparing patient’s self-reporting of pain tolerance and the pain experience and the practical aspects that would support the potential for clinical relevance. Additional mining of the data remains possible. The arbitrary determination of “5” on the numerical visual analog scales as the midpoint did not represent the actual mean or median responses of the subjects. The medical information collection forms used in this report were not validated. The reliability of the responses to the pain tolerance and impact testing was on a small subset of asymptomatic men who returned for repeat testing. However, the men’s results were consistent on the pain experience testing at both time intervals. The data input would ideally include input from more and different practice environments. Additional anatomical regions should be considered. There may be usefulness in establishing the issue of pain tolerance when caring for patients with osteoarthritis and pain following orthopedic injuries and surgery [1, 38, 39].

This multifaceted patient pain profile provided information not otherwise available from existing pain assessments methods. The expectation that there would be a potential clinical value was realized from this study of 359 knee patients and 193 adjunct study subjects of both genders [36]. There was potential clinical value in patient selection for various therapies as well as subsequent pain management. This would be important quantitative information if the future patient was like one of the 28% identified in the study that had a discrepancy between their opinion of pain tolerance and the results of their impact test experience. This subset experienced more pain than their opinion would have predicted. This pain assessment instrument provides an opportunity to identify such a patient prior to therapeutic intervention. It may be explained that it is probable that surgery would be a more painful experience than the small lead ball drop on the finger nail or knee used in this pain assessment. This patient may want to be evaluated at a pain clinic prior to any intervention. If a patient so identified was to have surgery, their pain control is likely to require more medication that those who did not this type of preoperative assessment results.

The physical instrument was inexpensive as it was constructed from readily available local hardware store materials. It has no moving parts to malfunction. The method provided a uniform impact with same lead weight dropped from standardized distances utilizing gravity force. The instrument was not subject to wear nor had the necessity of calibration (gravity was the source) or service.

The method proved practical and efficient to administer. The method including the impact testing experience took only an average of 5 min. The examiner did not require any special qualifications, knowledge or training as they follow simple directions and record the patient’s responses without interpretation. Entry of the information into the computer database was uncomplicated as the paper form layout matched the computer page design.

The choice of the finger nail and the patella proved to be ideal because of their relative sensibility and accessibility. The testing method provided a spectrum of responses available for analysis, but without an intolerable pain experience. There were no deleterious physical effects on the person’s body parts from this testing.

There was clinically relevant information gleaned from this study that was not otherwise available. Statistical analyses were used to find the mean pain tolerance and mean pain experienced, allowing for comparison between groups. The data showed that asymptomatic patients had a significantly higher self-reported pain tolerance than symptomatic patients, while collectively the pain they experienced during the finger test was no different. This information is clinically relevant as physicians would be aware that patients presenting with knee problems would have the accompanying potential to report their pain tolerance higher than if they had no problem while being no different in responses to the uniform impact testing than those who are asymptomatic. Although using the arbitrary midpoint showed that there were discrepancies in some patients’ tolerance versus pain experienced on uniform impact testing, the same findings were not found when taking the mean of the groups into consideration.

There was clinical value in that this study showed gender differences. Gender differences have been reported on asymptomatic gender cohorts when physical examination and plain film X-rays were compared [36]. As might have been expected the clinical knee patients were slightly more sensitive to the impact test on the affected knee than the opposite asymptomatic knee. This information should be helpful to a physician evaluating a patient with knee pain.

This study provides clinical information critical to pain management. Studies on pain management in the orthopedic literature report only the present intensity of pain the patient is experiencing. The methods rarely include a preoperative pain assessment bench mark [40, 41]. One such report on patients undergoing total knee replacement performed a visual analog scale and other psychometric assessments [40]. This study showed that subjective assessment of pain intensity had predictive value for post-operative pain and its duration. Reports that include pain management are advocating outpatient knee ligament reconstruction, as most patients were satisfied with the arthroscopic method and the pain management [41, 42]. Still up to 10% of these patients are not satisfied with their pain management [41, 42]. Perhaps a preoperative pain assessment tool reported herein would have identified those with potential for dissatisfaction based upon prior pain tolerance testing evidence. The recognition of such a patient profile could have modified pain management affording comfort. One report measured many preoperative clinical factors and their relationship to post-operative pain, but did not include a preoperative pain assessment [43]. It has been shown that post-operative recall of preoperative pain is not accurate in patients having total knee replacement [44]. This novel instrument and method would provide a valuable means of reminding the total knee patient of their preoperative condition and thus avoiding perception of an adverse post operative course.

There was clinical value to this method as the information was readily available for review on a computer screen by the patient in conjunction with the health care provider. Both the patient and the surgeon have the same data upon which to collaborate while making clinical decisions. The patient may view their responses concerning the amount of present pain, pain tolerance and the impact testing. The software provides instantaneous opportunity for the patient to view their responses with or without comparison to others like them, all subjects, all patients, all asymptomatic volunteers, all knee patients and/or gender specific and how they compared to others. A particular patient’s profile can be shared with them in the context of other patient profiles to aid in patient selection and postoperative pain management. The printing of the results may be helpful for the patient’s subsequent review. Consistency and inconsistencies of their responses would be pointed out to the patient especially if the rating of tolerance was high and the experience to the impact tests showed greater pain than the average of others like them. This type of patient may use this information to assist them in determining their therapeutic choices and the requirements for alleviation of their pain. They may want to consider a pain management clinic consultation for evaluation prior to therapeutic intervention. The surgeon insight is expanded beyond the patient’s reporting of pain magnitude. The physician gains insight not otherwise available to assess the patient’s subsequent potential response to the pain of surgery and peri-surgical management. This pre-intervention information has the potential to avoid patient dissatisfaction with the outcome of the therapy in regards to pain relief. In addition, improved patient selection in this regard may reduce medical malpractice occurrence. It seems this novel pain assessment method would have a standardization application to appropriately judge the outcome of surgical intervention for clinical trials and outcome studies. When sufficient data become available by this method, there would be the potential for outcome prediction concerning any given patient or treatment method.

There is a potential for practical use in pain management and clinics devoted to such.

There is the potential of clinical value in athletic medicine. This type of evaluation when established prior to the season and or injury would provide valuable in patient management during the season. The information obtained from a potential patient athlete by this method may be of help during the subsequent peri-operative care. Although designed for orthopedics and sports injuries, this method has potential for wider application to other medical conditions. This method certainly is more practical and less expensive than recent reports of the use of MRI as a means of assessment [45].

This pain assessment method has potential for research purposes. It seems this method reported herein would be easier and faster to administer than the Patient Reported Outcome Measurement Information System (PROMIS) [28]. That program includes 120 questions specifically about pain, in addition to hundreds relating to other aspects of their quality of life, such as anxiety, depression and fatigue. That computerized test aims to quantify pain, yet by subjective responses. More than 1,000 researchers have volunteered to try the new tool. It appears to be lacking the comparison of opinion and an actual experience. “One main goal of the PROMIS initiative is to develop a set of publicly available computerized adaptive tests for the clinical research community.”

The patient pain profile described herein has the potential give both the patient and the physician a better understanding of the condition being evaluated. The abundance of information gathered in the patient pain profile should be relevant for practical clinical use as well as research.

## References

[1] Kane RL, Bershadsky B, Lin WC, Rockwood T, Wood K (2002). Efforts to standardize the reporting of pain. J Clin Epidemiol.

[2] Bradham DD (1994). Outcomes research in orthopedics: history, perspectives, concepts, and future. Arthroscopy.

[3] Dworkin RH, Nagasako EM, Galer BS, Turk DC, Melzack R (2001). Assessment of neuropathic pain. Handbook of Pain Assessment.

[4] Herr KA, Mobily PR, Kohout FJ, Wagenaar D (1998). Evaluation of the Faces Pain Scale for use with the elderly. Clin J Pain.

[5] Hicks CL, von Baeyer CL, Spafford PA, van Korlaar I, Goodenough B (2001). The Faces Pain Scale-Revised: toward a common metric in pediatric pain measurement. Pain.

[6] McCaffery M, Beebe A (1989). Pain: clinical manual for nursing practice.

[7] Scott J, Huskisson EC (1976). Graphic representation of pain. Pain.

[8] Wilkie D, Lovejoy N, Dodd M, Tesler M (1990). Cancer pain intensity measurement: concurrent validity of three tools--finger dynamometer, pain intensity number scale, visual analogue scale. Hosp J.

[9] Bellamy N, Buchanan WW, Goldsmith CH, Campbell J, Stitt LW (1988). Validation study of WOMAC: a health status instrument for measuring clinically important patient relevant outcomes to antirheumatic drug therapy in patients with osteoarthritis of the hip or knee. J Rheumatol.

[10] Phillips DM (2000). JCAHO pain management standards are unveiled. Joint Commission on Accreditation of Healthcare Organizations. JAMA.

[11] Doornberg JN, Ring D, Fabian LM, Malhotra L, Zurakowski D, Jupiter JB (2005). Pain dominates measurements of elbow function and health status. J Bone Joint Surg Am.

[12] Breivik H, Borchgrevink PC, Allen SM, Rosseland LA, Romundstad L, Hals EK, Kvarstein G (2008). Assessment of pain. Br J Anaesth.

[13] Bwilym SE, Pollard TCB, Carr AJ (2008). Understanding pain in osteoarthritis. JBJS.

[14] Dykstra DL, Curtis Bay R, Bliven KH, Snyder AR (2011). Upper Extremity Injury History, Current Pain Rating, and Health-Related Quality of Life in Female Softball Pitchers. USR.

[15] Garrett WE, Kaeding CC, ElAttrache NS, Xerogeanes JW, Hewitt MS, Skrepnik NV, Papilion JD (2011). Novel drug OMS103HP reduces pain and improves joint motion and function for 90 days after arthroscopic meniscectomy. Arthroscopy.

[16] Jensen MP, Gammaitoni AR, Olaleye DO, Oleka N, Nalamachu SR, Galer BS (2006). The pain quality assessment scale: assessment of pain quality in carpal tunnel syndrome. J Pain.

[17] Leresche L (2011). Defining gender disparities in pain management. Clin Orthop Relat Res.

[18] Lundblad H, Kreicbergs A, Jansson KA (2008). Prediction of persistent pain after total knee replacement for osteoarthritis. J Bone Joint Surg Br.

[19] McGeary DD, Mayer TG, Gatchel RJ (2006). High pain ratings predict treatment failure in chronic occupational musculoskeletal disorders. J Bone Joint Surg Am.

[20] Melzack R, Katz J, McMahon SB, Koltzenburg M (2006). Pain assessment in adult patients. Wall and Melzack’s Textbook of Pain.

[21] Riddle DL, Wade JB, Jiranek WA, Kong X (2010). Preoperative pain catastrophizing predicts pain outcome after knee arthroplasty. Clin Orthop Relat Res.

[22] Vranceanu AM, Barsky A, Ring D (2009). Psychosocial aspects of disabling musculoskeletal pain. J Bone Joint Surg Am.

[23] Kawamata M, Takahashi T, Kozuka Y, Nawa Y, Nishikawa K, Narimatsu E, Watanabe H (2002). Experimental incision-induced pain in human skin: effects of systemic lidocaine on flare formation and hyperalgesia. Pain.

[24] Pedersen JL, Kehlet H (1998). Secondary hyperalgesia to heat stimuli after burn injury in man. Pain.

[25] Streff A, Kuehl L, Michaux G, Anton F (2009). Differential physiological effects during tonic painful hand immersion tests using hot and ice water. Euro J Pain.

[26] Werner MU, Duun P, Kraemer O, Lassen B, Kehlet H (2003). Arthroscopic knee surgery does not modify hyperalgesic responses to heat injury. Anesthesiology.

[27] Werner MU, Duun P, Kehlet H (2004). Prediction of postoperative pain by preoperative nociceptive responses to heat stimulation. Anesthesiology.

[28] Cella D, Yount S, Rothrock N, Gershon R, Cook K, Reeve B, Ader D (2007). The Patient-Reported Outcomes Measurement Information System (PROMIS): progress of an NIH Roadmap cooperative group during its first two years. Med Care.

[29] Herr K, Spratt KF, Garand L, Li L (2007). Evaluation of the Iowa pain thermometer and other selected pain intensity scales in younger and older adult cohorts uning controlled clinical pain; a preliminary study. Pain Med.

[30] http://medical-dictionary.thefreedictionary.com/profile.

[31] Granot M, Lowenstein L, Yarnitsky D, Tamir A, Zimmer EZ (2003). Postcesarean section pain prediction by preoperative experimental pain assessment. Anesthesiology.

[32] Hapidou EG, DeCatanzaro D (1992). Responsiveness to laboratory pain in women as a function of age and childbirth pain experience. Pain.

[33] Rawal N (2007). Postoperative pain treatment for ambulatory surgery. Best Pract Res Clin Anaesthesiol.

[34] Robinson KP, Wagstaff KJ, Sanghera S, Kerry RM (2014). Postoperative pain following primary lower limb arthroplasty and enhanced recovery pathway. Ann R Coll Surg Engl.

[35] Kurdi MS, Theerth KA, Deva RS (2014). Ketamine: Current applications in anesthesia, pain, and critical care. Anesth Essays Res.

[36] Johnson LL, van Dyk GE, Green JR, Pittsley AW, Bays B, Gully SM, Phillips JM (1998). Clinical assessment of asymptomatic knees: comparison of men and women. Arthroscopy.

[37] http://www.hhs.gov/ohrp/humansubjects/guidance/45cfr46.html#46.101.

[38] Ekman EF, Koman LA (2005). Acute pain following musculoskeletal injuries and orthopaedic surgery: mechanisms and management. Instr Course Lect.

[39] Gwilym SE, Pollard TC, Carr AJ (2008). Understanding pain in osteoarthritis. J Bone Joint Surg Br.

[40] Brander VA, Stulberg SD, Adams AD, Harden RN, Bruehl S, Stanos SP, Houle T (2003). Predicting total knee replacement pain: a prospective, observational study. Clin Orthop Relat Res.

[41] Brown DW, Curry CM, Ruterbories LM, Avery FL, Anson PS (1997). Evaluation of pain after arthroscopically assisted anterior cruciate ligament reconstruction. Am J Sports Med.

[42] Williams JS, Wexler G, Novak PJ, Bush-Joseph CA, Bach BR, Badrinath SK (1998). A prospective study of pain and analgesic use in outpatient endoscopic anterior cruciate ligament reconstruction. Arthroscopy.

[43] Asano H, Muneta T, Shinomiya K (2002). Evaluation of clinical factors affecting knee pain after anterior cruciate ligament reconstruction. J Knee Surg.

[44] Lingard EA, Wright EA, Sledge CB (2001). Pitfalls of using patient recall to derive preoperative status in outcome studies of total knee arthroplasty. J Bone Joint Surg Am.

[45] Wager TD, Atlas LY, Lindquist MA, Roy M, Woo CW, Kross E (2013). An fMRI-based neurologic signature of physical pain. N Engl J Med.

